# Assessing the performance of genetic risk score for stratifying risk of post-sepsis cardiovascular complications

**DOI:** 10.3389/fcvm.2023.1076745

**Published:** 2023-02-28

**Authors:** Brian McElligott, Zhuqing Shi, Andrew S. Rifkin, Jun Wei, S. Lilly Zheng, Brian T. Helfand, Jonathan S. H. Woo, Jianfeng Xu

**Affiliations:** ^1^Program for Personalized Cancer Care, NorthShore University HealthSystem, Evanston, IL, United States; ^2^Department of Surgery, NorthShore University HealthSystem, Evanston, IL, United States; ^3^Pritzker School of Medicine, University of Chicago, Chicago, IL, United States; ^4^Department of Medicine, NorthShore University HealthSystem, Evanston, IL, United States; ^5^Neaman Center for Personalized Medicine, NorthShore University HealthSystem, Evanston, IL, United States

**Keywords:** sepsis, myocardial infarction, ischemic stroke, venous thromboembolism, genetic risk score, polygenic

## Abstract

**Background:**

Patients with sepsis are at increased risk for cardiovascular complications, including myocardial infarction (MI), ischemic stroke (IS), and venous thromboembolism (VTE). Our objective is to assess whether genetic risk score (GRS) can differentiate risk for these complications.

**Methods:**

A population-based prospective cohort of 483,177 subjects, derived from the UK Biobank, was followed for diagnosis of sepsis and its complications (MI, IS, and VTE) after the study recruitment. GRS for each complication was calculated based on established risk-associated single nucleotide polymorphisms (SNPs). Time to incident MI, IS, and VTE was compared between subjects with or without sepsis and GRS risk groups using Kaplan–Meier log-rank test and Cox-regression analysis.

**Results:**

During an average of 12.6 years of follow-up, 10,757 (2.23%) developed sepsis. Patients with sepsis had an overall higher risk than non-sepsis subjects for each complication, but the risk differed by time after a sepsis diagnosis; exceedingly high in short-term (0–30 days), considerably high in mid-term (31 days to 2 years), and reduced in long-term (>2 years). Furthermore, in White subjects, GRS was a significant predictor of complications, independent of sepsis and other risk factors. For example, GRS_MI_ further differentiated their risk in patients with sepsis; 3.49, 4.73, and 9.03% in those with low- (<0.5), intermediate- (0.5–1.99), high- GRS_MI_ (≥2.0), *P_trend_* < 0.001.

**Conclusion:**

Risk for post-sepsis cardiovascular complications differed considerably by time after a sepsis diagnosis and GRS. These findings, if confirmed in other ancestry-specific populations, may guide personalized management for preventing post-sepsis cardiovascular complications.

## Introduction

Sepsis, a life-threatening organ dysfunction caused by a dysregulated host response to infection, is a major public health concern globally (1). It was estimated 48.9 million sepsis cases and 11 million sepsis related deaths worldwide in 2017 (2). While short-term sepsis mortality is decreasing, mid- to long-term sepsis mortality has remained high (3–5). Survivors of severe sepsis had higher rates of cardiovascular complications, including myocardial infarction (MI), ischemic stroke (IS) and venous thromboembolism (VTE) (6–10). The increased risk of these post-sepsis cardiovascular complications has been attributed to a variety of pathophysiologic mechanisms, including immunoparalysis, depression of ventricular function, arrhythmia, organ ischemia related to increased oxygen demand, procoagulant changes in the blood, endothelial dysfunction, impaired adrenergic response at the cardiomyocyte level, cardiomyocyte apoptosis, mitochondrial dysfunction and accelerated atherosclerosis (11–14).

Multiple clinical risk factors for post-sepsis cardiovascular complications have been identified, including older age, valvular heart diseases, coagulopathy, hypertension, peripheral vascular diseases, pulmonary circulation disorders, renal failure, and rheumatoid arthritis/collagen vascular diseases (9, 10, 15, 16). These risk factors can be used to identify a subpopulation of patients with sepsis that could be targeted to reduce the risk of complications. However, to date, no inherited risk factors for post-sepsis cardiovascular complications have been reported. With the identification of multiple common single nucleotide polymorphisms (SNPs) that are associated with corresponding increased risk of MI, IS, and VTE in general populations from genome-wide association studies (GWAS) (17–19), we hypothesized that polygenic risk score based on these risk-associated SNPs can identify patients at higher risk for developing short-, mid- and long-term post-sepsis cardiovascular complications. The objective of this study was to test this hypothesis in a large population-based prospective cohort.

## Methods

Subjects in this study were derived from the UK Biobank (UKB), a population-based study of approximately 488,000 volunteers from the United Kingdom aged 40–69 years at the time of recruitment (20). Extensive phenotypic information at study recruitment is available for each participant in the UKB, including disease diagnosis and risk factors for atherosclerotic cardiovascular disease (ASCVD). Health-related conditions during the follow-up were also available through linking to the National Health Service, including hospital inpatient data, coded primary care data, cancer and death registry data. In addition, genome-wide SNP genotyping data from either the UK Biobank Axiom array (for ~90% subjects) or UK BiLEVE array (for ~10% subjects) were available to all subjects.

A cohort of subjects without a diagnosis of sepsis at time of the UKB recruitment was identified (*N* = 472,420). These subjects were followed for incident MI, IS, and VTE (the last date of data access was 12/01/2021). The criteria used in the study by Jolley et al. were used to define sepsis (21). Specifically, subjects with International Classification of Diseases, 10th revision (ICD-10) codes of A40 (A40.0, A40.1, A40.2, A40.3, A40.8, and A40.9), A41 (A41.0, A41.1, A41.2, A41.3, A41.4, A41.5, A41.8, and A41.9), R65.2 were considered as sepsis. In addition, ICD-10 codes (I21, I22, I23, I24.1, and I25.2), ICD-9 codes (410, 411, 412, and 429.79) were used to identify MI, and ICD-10 (I63, I64), ICD-9 (434 and 436) for used to identify IS. For VTE, the criteria described by Klarin et al. (22) were used. Subjects were defined as a VTE case based on at least one of the following criteria: (1) VTE (deep vein thrombosis and pulmonary embolism) ascertained at baseline by self-report; (2) Hospitalization for ICD-10 Codes I80.1, I80.2, I82.2, I26.0, or I26.9; and (3) Hospitalization for Office of Population and Censuses and Survey-4 (OPCS-4) Procedures Codes L79.1 or L90.2. Hospitalization for ICD-10 Codes I81, I82.0, I80.0, I80.3, I80.8, I80.9, or D68 were excluded.

We calculated genetic risk score (GRS), an odds ratio (OR)-weighted and population-standardized polygenic risk score, for each trait of interest as follows (23). GRS was calculated as


$$\begin{array}{l} GRS=\prod_{i=1}^{n}\frac{{\mathrm{OR}}_{i}^{gi}}{Wi}\\ W_{i}={f_{i}}^{2}O{R_{i}}^{2}+2f_{i}\left(1-f_{i}\right)OR_{i}+{\left(1-f_{i}\right)}^{2} \end{array}$$

where, $g_{i}$ stands for number of risk alleles of SNP *i* in an individual (0, 1, or 2), *OR_i_* stands for the OR of SNP i estimated from external studies, and *f_i_* stands for the risk allele frequency of SNP i based on gnomAD (Non-Finnish European population). Because GRS is population standardized, its value can be interpreted as relative risk to the general population regardless number of SNPs used in the calculation. For example, a GRS of 1.5 indicate a 1.5-fold increased risk for a disease compared to the general population.

Considering that most of the established risk-associated SNPs for MI, IS, and VTE were from GWAS of European descent (17, 22, 24), GRS was only calculated for self-reported White subjects in the study. For each disease, independent risk-associated SNPs, defined as those meeting GWAS significance level (*p* < 5E-08) and pairwise linkage disequilibrium (LD) measurement of *r*^2^ < 0.2 were used. For SNPs with *r*^2^ < 0.2, the SNP with the highest *W* was used. The risk-associated SNPs for MI (78 SNPs), IS (30 SNPs), and VTE (22 SNPs) are presented in Supplementary Tables S1–S3, respectively, together with their allele frequency, OR, and references (17–19, 22, 24–26). All but two risk-associated SNPs are common, with minor allele frequency > 5%.

For patients with sepsis, time to adverse cardiovascular event was determined from diagnosis of sepsis to diagnosis of adverse cardiovascular event. For patients without sepsis, time to adverse cardiovascular event was determined from study recruitment to diagnosis of adverse cardiovascular event. The last day of the follow-up was 12/01/2021. The difference in time to adverse cardiovascular event by the status of sepsis and by GRS was tested using the log-rank test. Time to adverse cardiovascular event after a sepsis diagnosis was further grouped into short-term (0–30 days), mid-term (31 days to 2 years), and long-term (>2 years) (4). For each time frame, only subjects who were at risk for complications were included in the analysis and subjects died from any causes were censored. Furthermore, association of complications with sepsis and GRS was also tested using a multivariable Cox proportional hazards regression analysis, adjusting for age, gender, race, 10-year ASCVD risk based on pooled cohort equation, and genetic background (the top 10 principal components provided by the UKB) (27). All statistical analysis was performed using R (v4.0.5; R Core Team 2021).

## Results

Over an average of 12.6 years of follow-up out of a total of 483,177 participants who did not have a sepsis diagnosis at the study recruitment, 10,757 (2.2%) developed sepsis. Older age, male gender, and higher 10-year ASCVD risk were significantly associated with higher probability of incident sepsis, all *p* < 0.001 (Supplementary Table S4). The risk was slightly higher in subjects of self-reported White (2.24%) than that self-reported Black (2.10%), Asian (1.94%), and other (2.00%). The different proportions were not statistically significant, χ^2^ = 7.43, degree of freedom (df) = 3, *p* = 0.06.

Compared to subjects without a sepsis diagnosis, patients with sepsis had higher rates of developing MI, IS, VTE, or composite complications (any of these three complications), respectively (Table 1). Hazard ratio (HR) and 95% confidence interval (CI) was 1.18 (1.07–1.31), 1.45 (1.26–1.67), 3.41 (3.11–3.73), and 1.88 (1.77–2.00), respectively, for MI, IS, VTE, and composite complications, all *p* < 0.001. These risk estimates were obtained after adjusting covariates (age at recruitment, gender, race, 10-year ASCVD risk, and genetic background).

**Table 1:** Major cardiovascular complications in the sepsis cohort of the UK Biobank.

	Diagnosis of sepsis			
Major complications	Yes (*N* = 10,757)	No (*N* = 472,420)	HR^1^ (95% CI)	*p*-Value^1^
Incident myocardial infarction (MI), No. (%)	506 (4.7)	15,012 (3.18)	1.18 (1.07–1.31)	1.39E-03
Incident ischemic stroke (IS), No. (%)	271 (2.52)	6,543 (1.38)	1.45 (1.26–1.67)	1.84E-07
Incident venous thromboembolism (VTE), No. (%)	638 (5.93)	8,102 (1.71)	3.41 (3.11–3.73)	6.20E-150
Any incident MI, IS, and VTE, No. (%)	1,330 (12.36)	28,052 (5.94)	1.88 (1.77–2.00)	1.94E-85

^1^HR adjusted for age, gender, race, 10-year ASCVD Risk by PCE, and genetic background. Sepsis cases before recruitment were excluded, MI/IS/VTE cases before sepsis were excluded.

Time to develop these cardiovascular events (MI, IS, VTE, and composite complications) in subjects with or without sepsis is presented in Supplementary Figures S1A–D. Subjects with a sepsis diagnosis had significantly increased risks for these cardiovascular events compared to those without, all *p* < 0.001. When examining the three time frames after a sepsis diagnosis, the risk for these complications was exceedingly high during the short-term, considerably high during the mid-term, but reduced during the long-term (Table 2). For example, compared to subjects without sepsis, HR (95% CI) for MI was 137.95 (95.16–199.99), 3.70 (3.06–4.48), 0.40 (0.33–0.48), respectively, in the short-, mid-, and long-term after a sepsis diagnosis, all *p* < 0.001. These risk estimates were obtained after adjusting covariates (age at recruitment, gender, race, 10-year ASCVD risk, and genetic background). It is noted that some estimates in the short-term were either not provided by the statistical modeling (VTE) or unreliable due to small numbers of events, especially among patients without sepsis (for example, 20 and 19 subjects with IS and VTE, respectively).

**Table 2:** Differential risk for cardiovascular complications by follow-up time period after a sepsis diagnosis.

	HR (95% CI), *p*-Value^1^		
Major complications	Short-term (0–30 days)	Mid-term (31 days–2 years)	Long-term (>2 years)
Incident myocardial infarction (MI)	137.95 (95.16–199.99), *p* = 4.52e-149	3.70 (3.06–4.48), *p* = 1.31e-41	0.40 (0.33–0.48), *p* = 1.60e-22
Incident ischemic stroke (IS)	>999 (17.86- > 999), *p* = 8.11e-03	4.92 (3.80–6.37), *p* = 2.08e-33	0.40 (0.31–0.53), *p* = 1.23e-10
Incident venous thromboembolism (VTE)	NA (NA-NA), *p* = NA	11.03 (9.26–13.12), *p* = 9.18e-161	0.63 (0.51–0.79), *p* = 4.12e-05
Any incident MI, IS, and VTE	>999 (>999- > 999), *p* = 6.50e-40	5.88 (5.23–6.61), *p* = 1.24e-192	0.46 (0.40–0.52), *p* = 7.30e-31

^1^HR adjusted for age, gender, race, 10-year ASCVD Risk by PCE, and genetic background. Sepsis cases before recruitment were excluded, MI/IS/VTE cases before sepsis were excluded.

**Figure 1:**
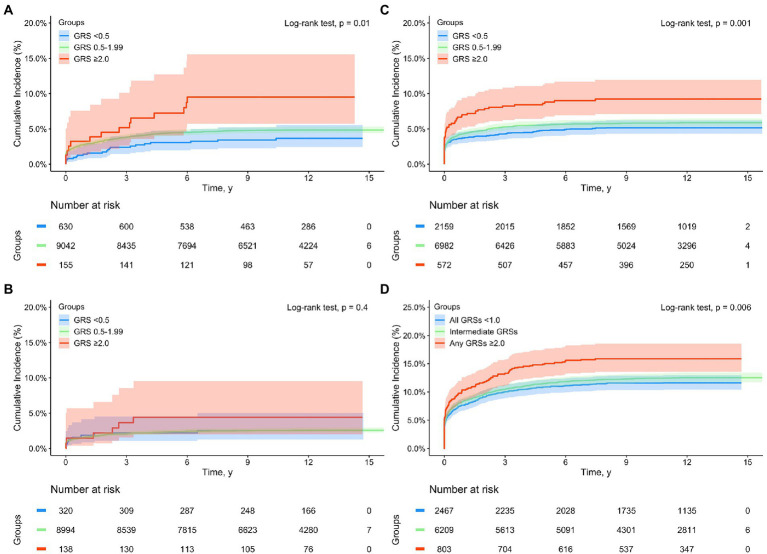
Kaplan–Meier analysis for time to incident cardiovascular complications from a sepsis diagnosis in subjects with a sepsis diagnosis in the UK Biobank (White subjects only) by GRS groups: **(A)** myocardial infarction (MI), **(B)** ischemic stroke (IS), **(C)** venous thromboembolism (VTE), and **(D)** composite complications (any of MI, IS, and VTE). Blue, green, and red lines denote subjects with GRS <0.5, 0.5–1.99, and ≥2.0, respectively.

When GRS was added in multivariate Cox-regression analyses among White subjects, both sepsis and disease-specific GRS (GRS_MI_, GRS_IS_, and GRS_VTE_, modeled as continuous variable) were significantly associated with complications during the entire follow-up, all *p* < 0.001 (Supplementary Table S5). For example, higher disease-specific GRS was associated with respective complication independent of sepsis status, HR was 1.93 (1.08–2.00), 1.26 (1.17–1.35), and 1.42 (1.37–1.47), respectively, for MI, IS, and VTE. Similar results were found in the Kaplan–Meier analysis for subjects stratified by sepsis status (yes and no) and GRS groups (<0.5, 0.5–1.99, and >2.0) (Supplementary Figures S2A–D). The differences in time to develop cardiovascular events among these six groups were statistically significant for each complication and for the composite complications, all *p* < 0.001. Except for MI, the cumulative complication event rates were consistently higher in subjects with sepsis than without sepsis, and in subjects with higher GRS risk category in those with or without sepsis. For MI, the cumulative rates in patients with sepsis who had low- or intermediate-GRS_MI_ were lower than that of non-sepsis patients with high-GRS_MI_ after ~6 years of follow-up. Among patients with incident sepsis, a dose–response of cardiovascular event rates with increasing GRS risk groups was found (Figure 1). The trend was statistically significant for MI, VTE and composite complications.

The estimated proportions of these cardiovascular events in subjects with or without sepsis, as well as in sepsis patients with low-, intermediate-, and high-GRS are presented in Table 3. Appreciable differences were found during the entire follow-up as well as in short-, mid-, and long-term. For example, the cumulative incident rate of MI was 3.17 and 4.69% in subjects with or without sepsis, *p* < 0.001. Among patients with sepsis, GRS_MI_ further differentiated their risk; 3.49, 4.73, and 9.03% in those with low-, intermediate-, high-GRS_MI_, *P_trend_* < 0.001.

**Table 3:** Rate of cardiovascular complications by follow-up time period after a sepsis diagnosis, race of white.

		No. (%) of complication after a sepsis diagnosis			
Major complications	No. (%) subjects	Entire follow-up	0–30 days	31 days–2 years	>2 years
Incident myocardial infarction (MI)					
Sepsis –	444,311 (97.76)	14,063/444,311 (3.17)	48/444,311 (0.01)	1,234/444,192 (0.28)	12,776/442,958 (2.88)
Sepsis +	10,182 (2.24)	478/10,182 (4.69)	179/10,182 (1.76)	158/9,994 (1.58)	141/9,836 (1.43)
GRS_MI_ < 0.5	630 (6.41)	22/630 (3.49)	4/630 (0.63)	7/626 (1.12)	11/619 (1.78)
GRS_MI_ 0.5–1.99	9,042 (92.01)	428/9042 (4.73)	167/9042 (1.85)	142/8866 (1.6)	119/8724 (1.36)
GRS_MI_ ≥ 2.0	155 (1.58)	14/155 (9.03)	3/155 (1.94)	4/152 (2.63)	7/148 (4.73)
Incident ischemic stroke (IS)					
Sepsis –	444,311 (97.76)	6,202/444,311 (1.4)	20/444,311 (0)	524/444,221 (0.12)	5,658/443,697 (1.28)
Sepsis +	10,182 (2.24)	257/10,182 (2.52)	108/10,182 (1.06)	91/10,065 (0.9)	58/9,974 (0.58)
GRS_IS_ < 0.5	320 (3.39)	8/320 (2.5)	3/320 (0.94)	4/317 (1.26)	1/313 (0.32)
GRS_IS_ 0.5–1.99	8,994 (95.15)	226/8994 (2.51)	94/8994 (1.05)	81/8892 (0.91)	51/8811 (0.58)
GRS_IS_ ≥ 2.0	138 (1.46)	6/138 (4.35)	2/138 (1.45)	1/136 (0.74)	3/135 (2.22)
Incident venous thromboembolism (VTE)					
Sepsis –	444,311 (97.76)	7,753/444,311 (1.74)	19/444,311 (0)	723/444,221 (0.16)	7,007/443,498 (1.58)
Sepsis +	10,182 (2.24)	604/10,182 (5.93)	290/10,182 (2.85)	212/9,883 (2.15)	102/9,671 (1.05)
GRS_VTE_ < 0.5	2,159 (22.23)	110/2159 (5.09)	60/2159 (2.78)	26/2097 (1.24)	24/2071 (1.16)
GRS_VTE_ 0.5–1.99	6,982 (71.88)	405/6982 (5.8)	188/6982 (2.69)	148/6787 (2.18)	69/6639 (1.04)
GRS_VTE_ ≥ 2.0	572 (5.89)	52/572 (9.09)	28/572 (4.9)	17/544 (3.12)	7/527 (1.33)
Any incident MI, IS, and VTE					
Sepsis –	444,311 (97.76)	26,503/444,311 (5.96)	88/444,311 (0.02)	2,437/444,153 (0.55)	23,969/441,716 (5.43)
Sepsis +	10,182 (2.24)	1,257/10,182 (12.35)	563/10,182 (5.53)	427/9,610 (4.44)	267/9,183 (2.91)
GRS_combined_: all GRS <1.0	2,555 (25.95)	292/2555 (11.43)	134/2555 (5.24)	98/2418 (4.05)	60/2320 (2.59)
GRS_combined_: remaining	6,450 (65.51)	791/6450 (12.26)	352/6450 (5.46)	269/6092 (4.42)	170/5823 (2.92)
GRS_combined_: any GRS ≥2.0	841 (8.54)	131/841 (15.58)	59/841 (7.02)	40/782 (5.12)	32/742 (4.31)

Sepsis cases before recruitment were excluded, MI/IS/VTE cases before sepsis were excluded.

## Discussion

In this large population-based cohort of subjects without sepsis at baseline, we confirmed the previous finding of higher cardiovascular complications (MI, IS, and VTE) among patients with sepsis. More importantly, we also obtained findings that GRS was significantly associated with these cardiovascular events in White subjects and this association was independent of sepsis and other ASCVD risk factors. Appreciable differences in the cumulative incident rate of cardiovascular complications by GRS risk groups were found. This finding from a prospective cohort, if confirmed in other ancestry-specific populations, may provide a useful tool for personalized prevention of post-sepsis cardiovascular complications.

To our knowledge, this study is the first polygenic risk score study of post-sepsis cardiovascular complications, and represents a novel clinical utility of established polygenic risk scores for cardiovascular diseases. The performance of polygenic risk scores for risk stratification of MI, IS, and VTE risk in the general population has been well established (28–31). The primary goal of this current study was to assess the performance of externally validated GRSs in the post-sepsis patients (17–19). We evaluated their utility to differentiate cumulative complication rate among patients with sepsis, adjusting for other known ASCVD risk factors. Obtained results from this study provided evidence that GRSs are useful and complementary tools for physicians to stratify risk for cardiovascular complications among patients with sepsis for personalized care. Practically, GRS is feasible and cost-effective with current low-coverage whole genome sequencing (lcWGS) and genome-wide SNP array technologies. These three GRSs, together with GRS for other diseases, can be calculated using a single low-cost assay (<$100 per patient) (32).

Recognizing that multiple polygenic risk score algorithms are available (33), we chose GRS as a method of choice in this study for the following considerations, (1) simple interpretation to facilitate clinical use (the value of GRS can be interpreted as relative risk to the general population regardless number of SNPs) (23), (2) risk-associated SNPs are well established in prior studies (17–19), (3) performance of various polygenic risk score algorithms are similar for cardiovascular diseases (33, 34), and (4) fewer number of SNPs for simplifying clinical laboratory regulation (compared with millions of SNPs in other PRSs).

The finding of reduced risk in the long-term (>2 years) after a sepsis diagnosis when compared to subjects without a sepsis diagnosis was unexpected. It is possible that this finding was confounded by potential bias such as differential mortalities and other unmeasured risk factors between subjects with or without sepsis. The effect of such bias, however, is likely small due to our prospective study design and analytical approach. Subjects with or without sepsis were analyzed the same way where only those at risk for cardiovascular complications after 2 years were included and those died of any cause were censored. Furthermore, a competing risk survival analysis that taking mortality into consideration provided similar finding (Supplementary Table S6). One hypothesis is that patients with sepsis who are at increased risk for cardiovascular complications were trigged by sepsis and developed immediately after sepsis (short- and mid-term), and the remaining patients with sepsis were at lower risk for these complications. These results suggest prevention of post-sepsis cardiovascular complications is more important in the short- and mid-term after sepsis. This finding and hypothesis should be confirmed and tested in other studies.

Results from this study may have potential clinical implications. GRS can be used to supplement other clinical variables and biomarkers such as troponin and d-dimer to better predict risk for cardiovascular complications (35–38). GRS, especially GRS_VTE_, can also be used to aid discussion about benefits and harms of anticoagulation (39). Finally, for sepsis patients with GRS ≥2 for MI, IS, or VTE, clinicians may consider increased surveillance of these complications. Further clinical studies will be required to determine optimal preventive approaches for high-risk patients.

This study has several strengths. First, a large number of patients with sepsis diagnosis (*n* = 10,182) were included in the study. Second, the study includes long-term outcomes with an average of 12.6 years follow-up. Third, our study included a separate risk of MI, IS, VTE as well as a composite of MI, IS, VTE in patients with sepsis unlike previous genetic studies focusing on a single disease in general patient populations.

The study also has limitations. While the study included subjects from multiple ancestral backgrounds, analysis of GRS was limited to White. This is due to the fact that most known risk-associated SNPs for MI, IS, VTE were identified in subjects of European descents and that ~94% of study participants in UK Biobank are White. The racial disparity has been a major limitation for many genetic studies including our study. Our results should be validated in other ancestral populations. Furthermore, due to challenges of accessing detailed clinical data at time of sepsis diagnosis, other clinical risk factors such as valvular heart diseases, coagulopathy, peripheral vascular diseases, pulmonary circulation disorders, and rheumatoid arthritis/collagen vascular diseases, and cardiovascular drug use (with the exception of antihypertensive use) were not adjusted for in estimating incident rates of cardiovascular complications. Additional studies with detailed clinical variables are needed for more comprehensive analyses. Finally, high HR for the probability of the MI in the first 30 days after sepsis was likely confounded by the type 2 MI.

In conclusion, these findings suggest potential clinical utility of disease-specific GRS in cardiovascular risk stratification among White individuals diagnosed with sepsis, potentially improving personalized prevention of adverse cardiovascular outcomes. Further studies involving participants with various ancestral and ethnical backgrounds are needed.

## Data availability statement

The datasets presented in this article are not readily available because the data was accessed through a Material Transfer Agreement in the UK Biobank under Application Reference Number: 50295. Requests to access the datasets should be directed to JX, jxu@northshore.org.

## Ethics statement

The studies involving human participants were reviewed and approved by North West – Haydock Research Ethics Committee (REC reference: 16/NW/0274; IRAS project ID: 200778). Data from the UK Biobank was accessed through a Material Transfer Agreement under Application Reference Number: 50295. This study was performed in accordance with the Declaration of Helsinki. The patients/participants provided their written informed consent to participate in this study.

## Author contributions

JSHW and JX: concept and design. ZS and JW: data analysis. BM, JSHW, and JX: manuscript draft. BM, ZS, AR, JW, SLZ, BH, JSHW, and JX: critical revision of the manuscript for important intellectual content. JX and JSHW: supervision. All authors take responsibility for all aspects of the reliability and freedom from bias of the data presented and their discussed interpretation. All authors contributed to the article and approved the submitted version.

## Conflict of interest

NorthShore University HealthSystem has an agreement with GoPath Laboratories for genetic tests of polygenic risk score.

## Publisher’s note

All claims expressed in this article are solely those of the authors and do not necessarily represent those of their affiliated organizations, or those of the publisher, the editors and the reviewers. Any product that may be evaluated in this article, or claim that may be made by its manufacturer, is not guaranteed or endorsed by the publisher.

## Supplementary material

SUPPLEMENTARY FIGURE S1 Kaplan-Meier analysis for time to incident cardiovascular complications from a sepsis diagnosis in the UK Biobank: **(A)** myocardial infarction (MI), **(B)** ischemic stroke (IS), **(C)** venous thromboembolism (VTE), and **(D)** composite complications (any of MI, IS, and VTE). Red and blue lines denote subjects with or without a sepsis diagnosis, respectively. The 1st, 2nd, 3rd, and 4th vertical dotted line indicates time from a sepsis diagnosis (1st month, and 1st, 2nd, and 3rd year, respectively).

SUPPLEMENTARY FIGURE S2 Kaplan-Meier analysis for time to incident cardiovascular complications from a sepsis diagnosis stratified by sepsis status and GRS risk groups in the UK Biobank (White subjects only): **(A)** myocardial infarction (MI), **(B)** ischemic stroke (IS), **(C)** venous thromboembolism (VTE), and **(D)** composite complications (any of MI, IS, and VTE). Lines with different colors denote subjects with or without a sepsis diagnosis, and three GRS risk groups (<0.5, 0.5-1.99, and ≥2.0).
