# Supplementary figures and images for: Assessing the performance of genetic risk score for stratifying risk of post-sepsis cardiovascular complications
Source: Front Cardiovasc Med. 2023 Feb 28;10:1076745. doi: 10.3389/fcvm.2023.1076745 (PMC10011112; doi:10.3389/fcvm.2023.1076745)

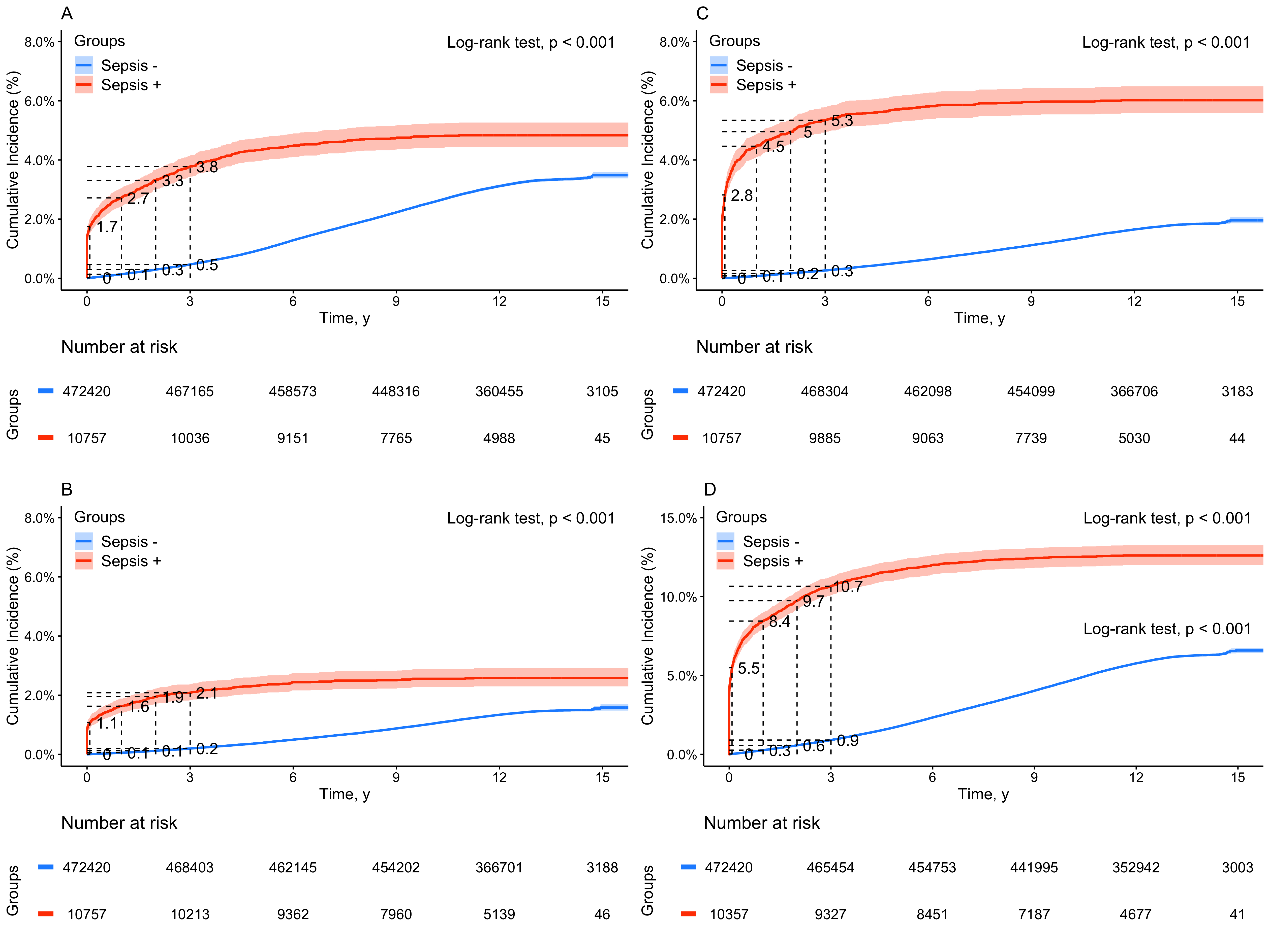

Supplement: SUPPLEMENTARY FIGURE S1 — Kaplan-Meier analysis for time to incident cardiovascular complications from a sepsis diagnosis in the UK Biobank: (A) myocardial infarction (MI), (B) ischemic stroke (IS), (C) venous thromboembolism (VTE), and (D) composite complications (any of MI, IS, and VTE). Red and blue lines denote subjects with or without a sepsis diagnosis, respectively. The 1st, 2nd, 3rd, and 4th vertical dotted line indicates time from a sepsis diagnosis (1st month, and 1st, 2nd, and 3rd year, respectively). [file Image_1.JPEG]

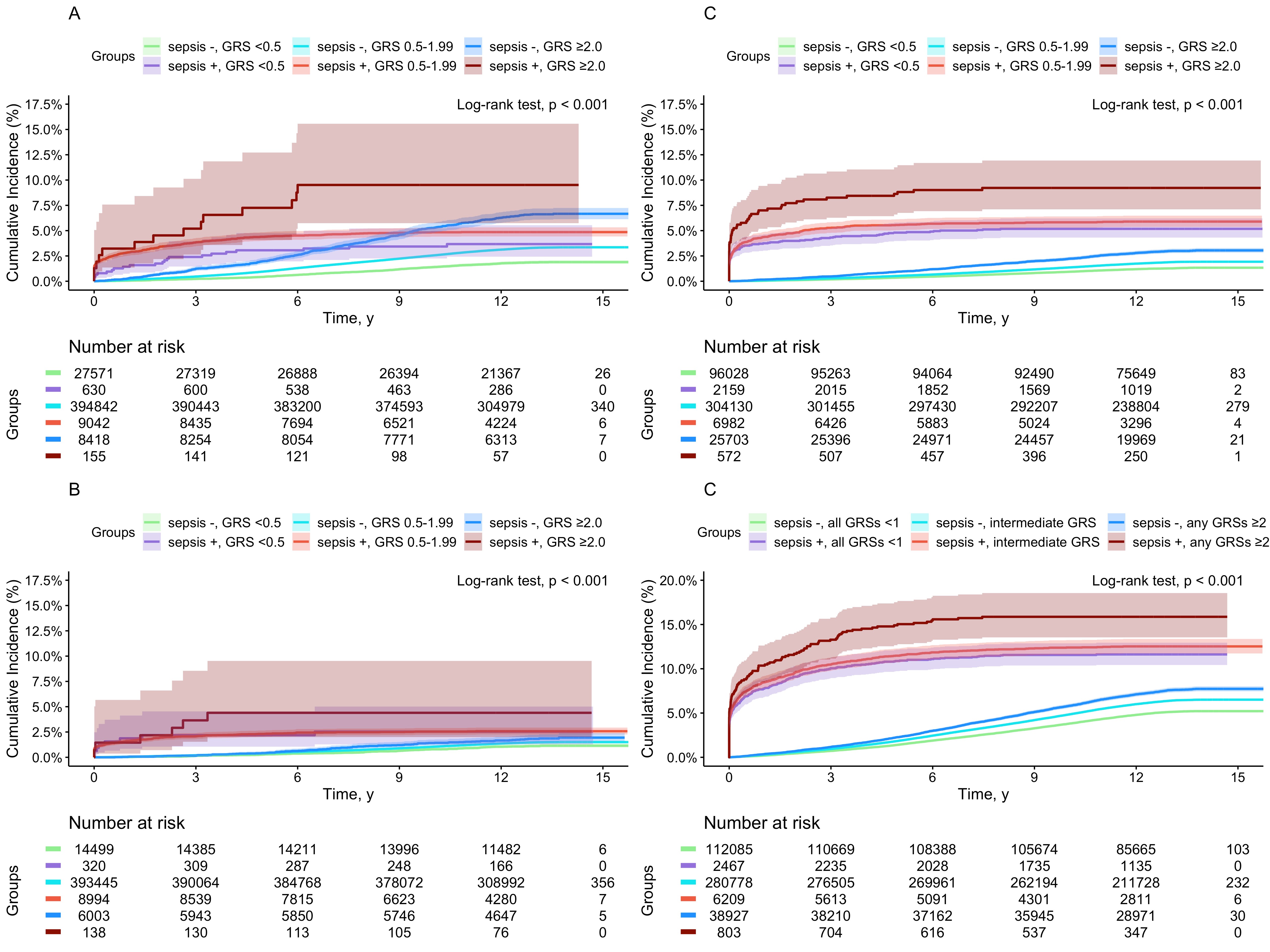

Supplement: SUPPLEMENTARY FIGURE S2 — Kaplan-Meier analysis for time to incident cardiovascular complications from a sepsis diagnosis stratified by sepsis status and GRS risk groups in the UK Biobank (White subjects only): (A) myocardial infarction (MI), (B) ischemic stroke (IS), (C) venous thromboembolism (VTE), and (D) composite complications (any of MI, IS, and VTE). Lines with different colors denote subjects with or without a sepsis diagnosis, and three GRS risk groups (<0.5, 0.5-1.99, and ≥2.0). [file Image_2.JPEG]
